# Expanding the Eco-collection of Methane-oxidizing Bacteria Inhabiting Rice Roots: Cultivation, Isolation, and Genomic Characterization of Isolates

**DOI:** 10.1264/jsme2.ME25012

**Published:** 2025-10-11

**Authors:** Fumika Oe, Rina Shinjo, Sachiko Masuda, Arisa Shibata, Ken Shirasu, Shun Hashimoto, Hisayuki Mitsui, Shusei Sato, Takeshi Watanabe, Susumu Asakawa

**Affiliations:** 1 Graduate School of Bioagricultural Sciences, Nagoya University, Furo-cho, Chikusa, Nagoya 464–8601, Japan; 2 RIKEN Center for Sustainable Resource Science, RIKEN-TRIP, 1–7–22 Suehiro-cho, Tsurumi, Yokohama, Kanagawa, 230–0045, Japan; 3 Graduate School of Life Sciences, Tohoku University, 2–1–1 Katahira, Aoba-Ku, Sendai, 980–8577, Japan

**Keywords:** Methane-oxidizing bacteria, Methanotrophs, Rice roots, Rice fields, Whole genome sequencing

## Abstract

Flooded rice fields are a major source of atmospheric methane, a strong greenhouse gas second only to carbon dioxide. Rice roots are one of the most important hotspots for methane oxidation in rice fields. However, limited information is available on the physiological and genomic characteristics of methane-oxidizing bacteria (MOB) inhabiting rice roots. In the present study, we isolated MOB from rice roots and characterized the strains phenotypically and genomically. We obtained 100 MOB-enriched cultures from the roots of three rice cultivars (*Oryza sativa* L. subsp. *japonica* cv. Nipponbare, *O. sativa* L. subsp. *indica* cv. Muha, and Tupa 121-3), in which twelve MOB isolates, two *Methylomonas* sp., three *Methylocystis* sp., and seven *Methylosinus* sp., were successfully purified. They showed different morphological features (types of flagellation) and colony formation potentials within the same group in some cases. A genome sequencing ana­lysis revealed variations in the number of genes or the clusters of methane monooxygenase, methanol dehydrogenase, and nitrogenase. The number of plasmid DNAs also differed among the strains. Four strains belonging to the genus *Methylomonas* or *Methylocystis* represented putative novel species based on their phenotypic and genotypic characteristics. The present study largely expanded the eco-collection of MOB cultures inhabiting rice fields and rice roots.

Rice is one of the most important staple food crops in the world, particularly in Asia (Khush, 1997; Muthayya *et al.*, 2014). The rice cultivation system contributes to global warming because flooded rice fields are a major anthropogenic source of atmospheric methane, a strong greenhouse gas second only to carbon dioxide (Intergovernmental Panel on Climate Change [IPCC], 2021). The amount of methane emitted from rice fields is measured from the difference between production by methanogens and consumption by methane oxidizers, mostly aerobic methane-oxidizing bacteria (MOB) (Conrad, 2007; Asakawa, 2021). Methanogens produce methane in the final step of the anaerobic decomposition of organic matter (Takai, 1970), and MOB utilize methane as the sole carbon and energy sources and convert it to CO_2_ (Hanson and Hanson, 1996). Aerobic MOB preferably inhabit aerobic compartments in rice fields, *e.g.*, flooded water, surface soils, and the rhizosphere (Gilbert and Frenzel, 1995; Eller and Frenzel, 2001; Eller *et al.*, 2005; Jia *et al.*, 2007; Takeda *et al.*, 2008; Dianou *et al.*, 2012; Shinjo *et al.*, 2023). Previous studies found that 61–97% and 0–94% of the methane produced in anoxic soil were oxidized in the surface soil layer of rice fields (Conrad and Rothfuss, 1991) and the rice rhizosphere (Frenzel *et al.*, 1992; Gilbert and Frenzel, 1995; Denier van der Gon and Neue, 1996), respectively, before being emitted via diffusion or ebullition into the atmosphere. Aerobic MOB have been suggested to reduce methane emissions from flooded rice fields.

Although aerobic MOB were traditionally classified into‍ ‍two groups: *Gammaproteobacteria* (type I) and *Alphaproteobacteria* (type II), novel MOB have since been found beyond these two classes, namely, acidophilic MOB in *Verrucomicrobiota* (type III) (Knief, 2015). Some *Mycobacterium* in *Actinomycetota* may also have the ability of perform methane oxidation (Kambara *et al.*, 2022; van Spanning *et al.*, 2022). Type I and II MOB play major roles in reducing methane emissions from rice fields (Henckel *et al.*, 2000; Macalady *et al.*, 2002). Type I and II MOB are characterized phylogenetically together due to their distinct structural, biochemical, and chemotaxonomic features: the intracellular membrane structure, carbon assimilation metabolic pathway, and number of carbons in major fatty acids (Hanson and Hanson, 1996). Many MOB strains have been isolated from rice fields; however, only six species of type I: *Methylogaea oryzae* (Geymonat *et al.*, 2011), *Methylomonas koyamae* (Ogiso *et al.*, 2012; Lee *et al.*, 2020), *Methylomagnum ishizawai* (Khalifa *et al.*, 2015; Frindte *et al.*, 2017),* Methyloterricola oryzae* (Frindte *et al.*, 2017), *Methylocucumis oryzae* (Pandit and Rahalkar, 2019), and *Methylococcus mesophilus* (Awala *et al.*, 2023), and one species of type II: *Methylocystis iwaonis* (Kaise *et al.*, 2023), have been characterized and described. MOB isolated from rice fields have been classified into types I and II in phylogenetics, but show diversity, *e.g.*, in their motility and types of methane monooxygenase. Although the genome sequences of several MOB isolates from rice fields have been elucidated apart from the identified strains, these strains have neither been deposited to any culture collections nor had their characteristics described in detail (Bao *et al.*, 2016; Rahalkar *et al.*, 2016; Ghashghavi *et al.*, 2019; Rahalkar *et al.*, 2019; Lee *et al.*, 2020; Yasuda *et al.*, 2020a; Zhu *et al.*, 2020; Rahalkar *et al.*, 2021; Mohite *et al.*, 2023). Therefore, current MOB resources are not satisfactory for a comprehensive understanding of the phenotypic and genotypic features of MOB inhabiting rice fields. Gorlach *et al.* (1994) introduced the concept of an “eco-collection”, referring to isolates collected from the main groups of populations, to uncover patterns or principles governing these populations. We follow this concept and need to expand the eco-collection of culturable MOB from rice fields.

Rice roots, where the influx of methane from soil to the atmosphere and efflux of O_2_ from the atmosphere to soil occur simultaneously, are one of the most important hotspots for methane oxidation and habitats for aerobic MOB. MOB populations in rice roots or on the root surface enumerated by the most probable number method were previously shown to be as high as 10^5^–10^7^ MOB g^–1^ dry weight of roots (Gilbert and Frenzel, 1995; Bosse and Frenzel, 1997; Gilbert *et al.*, 1998; Eller and Frenzel, 2001). A cultivation-independent ana­lysis showed that type II MOB were dominant in rice roots (Ikeda *et al.*, 2014; Hara *et al.*, 2022), while diverse and unknown type I MOB also inhabited rice roots (Horz *et al.*, 2001). Bao *et al.* (2014a) reported that type II MOB inhabited the vascular bundles and epidermal cells of rice roots and exhibited active methane oxidation and nitrogen fixation. Nevertheless, only three MOB strains have been isolated from rice roots, all belonging to type II MOB, and have been temporarily assigned to type II based on the sequences of their 16S rRNA genes (Takeda *et al.*, 2008; Bao *et al.*, 2016). No strain isolated from rice roots has been identified at the species level. These findings suggest that rice roots provide various habitats for a diverse MOB community and MOB work in their suited habitats in rice roots, which emphasizes the necessity of expanding the eco-collection of MOB and elucidating the diversity of their physiological and genomic features. These efforts will enable us to compare phenotypic and genetic features among the collections and expand our knowledge of their ecology. Therefore, we herein focused on rice roots as the source of novel MOB, including type I and II MOB, and aimed to obtain several MOB isolates in order to expand the collection of MOB inhabiting rice fields and reveal the whole genome sequences of these isolates. The present study provides a more detailed understanding of the eco-physiology of MOB in/on rice roots and promotes methane oxidation in/on rice roots for the mitigation of methane emissions from rice fields.

## Materials and Methods

### Rice root samples

Root samples were collected from three cultivars of rice plants: *Oryza sativa* L. subsp. *japonica* cv. Nipponbare, *O. sativa* L. subsp. *indica* cv. Muha, and Tupa 121-3. These plants were grown long term at a paddy field plot without nitrogen fertilizer in the Kashimadai experimental field, Tohoku University, Osaki, Miyagi, Japan (38°27'37"N, 141°5'3"E). The soil type of the rice field was Gleysol and detailed information on the field plot has been already reported by Bao *et al.* (2014b). Some characteristics of the soil were as follows: pH (H_2_O) 5.3; total nitrogen, 0.13%; total carbon, 1.3%; NH_4_^+^, 16.3‍ ‍mg kg^–1^ soil; available P (Truog), 69.4‍ ‍mg kg^–1^ soil; cation exchange capacity, 12.3‍ ‍cmol (+) kg^–1^ soil (Bao *et al.*, 2014b). Nipponbare was planted on 27th May 2020, and the roots were collected on 28th August at the heading stage. Nipponbare, Muha, and Tupa121-3 were planted on 24th May 2021, and the roots were obtained on 4th August, at which time Nipponbare was in the panicle initiation stage and Muha and Tupa 121-3 were in the heading stage. Soil attached to the roots was repeatedly washed off by tap water. The root samples (Nipponbare) collected in 2020 were stored at –30°C until used, while the samples (Nipponbare, Muha, and Tupa 121-3) collected in 2021 were stored at 4°C and used within 7 days. The roots were washed again by tap water and rinsed by distilled water before cultivation. All root samples were cut to approximately 1‍ ‍mm and pulverized by a mortar and pestle.

### Cultivation of MOB by the direct spread plate method

The direct spread plate method was performed to obtain MOB colonies directly from root samples. One gram of a root sample was suspended in 10‍ ‍mL of nitrate mineral salt (NMS) liquid medium (Whittenbury *et al.*, 1970) with a slight modification (KNO_3_, 1.0 g; MgSO_4_·7H_2_O, 1.0 g; Na_2_HPO_4_·12H_2_O, 0.72 g; KH_2_PO_4_, 0.26 g; CaCl_2_·2H_2_O, 0.26 g; EDTA-Fe, 38‍ ‍mg; Na_2_MoO_4_·2H_2_O, 0.26‍ ‍mg; trace metal solution [FeSO_4_·7H_2_O, 0.50‍ ‍g; ZnSO_4_·7H_2_O, 0.40 g; CuSO_4_·5H_2_O, 0.20 g; EDTA-2Na, 0.25 g; CoCl_2_·6H_2_O, 50‍ ‍mg; MnCl_2_·4H_2_O, 20‍ ‍mg; H_3_BO_3_, 15‍ ‍mg; NiCl_2_·6H_2_O, 10‍ ‍mg], 100‍ ‍μL; L^–1^ distilled water; pH 6.8) in a 34-‍ml screw-top test tube with a butyl rubber stopper (18‍ ‍mm i.d.×180‍ ‍mm; Sanshin Industrial) by vortexing for 1‍ ‍min. The suspension was further shaken at 120‍ ‍rpm at 4°C for 1‍ ‍h and diluted to 10^4^ or 10^6^ by the NMS liquid medium. The diluted solution was spread onto NMS agar plates and incubated at 25°C or 30°C under an approximately 50% (v/v) methane gas atmosphere in BBL Gaspak 100 Holding jars (Becton, Dickinson and Company). The colonies that formed on the plates were picked up and incubated in 10‍ ‍mL NMS liquid medium with 30% (v/v) methane gas in a 34-‍mL screw-top test tube with a butyl rubber stopper at 25°C. Methane consumption in a 0.1-mL sample from the headspace was assessed by a gas chromatograph with a flame ionization detector (GC-14B; Shimadzu). The sample was defined as a MOB culture when more than 5% methane was consumed.

### Cultivation of MOB by the enrichment culture method

Since the MOB population in the root samples may not have been sufficiently abundant to obtain MOB isolates using the direct spread plate method, the enrichment culture method was also performed in the present study. One gram of a root sample was inoculated into 25‍ ‍mL NMS liquid medium in a 250-mL bottle sealed with a butyl rubber stopper (DWK Life Science) and incubated at 25°C or 30°C under approximately 14% (v/v) methane gas and static conditions. Some of the cultures that showed a decrease in the methane concentration in the headspace were inoculated into fresh medium for the further enrichment of MOB. This enrichment step was repeated two to four times. MOB-enriched cultures were then serially diluted and spread onto NMS agar plates. The colonies that formed on the plates were picked up and inoculated into 10‍ ‍mL new NMS liquid medium with 30% (v/v) methane gas in a 34-mL screw-top test tube with a butyl rubber stopper at 25°C. The headspace measurement method and definition of the MOB culture were the same as those described above.

### Purification of MOB

We defined cultures that exhibited methane oxidation activity as MOB cultures. However, since the MOB cultures contained cells with different morphologies, possibly due to contamination by heterotrophic bacteria, further purification by two methods was conducted to obtain pure isolates of MOB: the repeated colony isolation and dilution-to-extinction methods.

Regarding colony isolation, a NMS agar plate (1.5% [w/v]) and NMS agar plate containing 0.05% yeast extract (Bowman, 2011) were used to distinguish MOB (slow growers) from contaminated heterotrophic bacteria (fast growers). A methanol medium (polypeptone, 10 g; yeast extract, 2.0 g; MgSO_4_·7H_2_O, 1.0 g; methanol, 0.5‍ ‍mL; L^–1^ distilled water; pH 7.0) agar plate (1.5% [w/v]) and NMS medium supplemented with 0.1% [w/w] sucrose were also used. In some cases, a NMS plate supplemented with gellan gum (0.4% [w/v]) was used instead of a NMS agar plate.

In the dilution-to-extinction method, we used three types of containers: 96-well plates, gas chromatograph vials, and 1,000-mL bottles sealed with a butyl rubber stopper. In the case of 96-well plates (Thermo Fisher Scientific), 20‍ ‍μL of serially diluted cultures was incubated with 180‍ ‍μL NMS liquid medium per well. The plates were placed in an AnaeroPack^TM^ rectangular jar (Mitsubishi Gas Chemical Company) and incubated at 25°C under an approximately 50% (v/v) methane gas atmosphere. When using gas chromatograph vials (30‍ ‍mm i.d.×70‍ ‍mm, Nichiden Rika Glass), 50‍ ‍μL of serially diluted cultures was incubated with 5 ml NMS liquid medium per vial at 25°C under approximately 30% (v/v) methane gas with shaking. When using 1,000-mL bottles sealed with a butyl rubber stopper (DWK Life Science), 1 ml of serially diluted cultures was incubated with 150‍ ‍mL NMS liquid medium per bottle at 30°C under approximately 50% (v/v) methane gas with shaking.

The purity of the isolates obtained was carefully checked by cell observations under a phase-contrast microscope (BX50; Olympus) and confirming the lack of growth of heterotrophic bacteria in nutrient-rich culture liquid media and agar plates (Luria–Bertani medium [Bertani, 1951], nutrient broth medium [Becton, Dickinson and Company], and methanol medium) for 1 month. MOB cultures were inoculated in 10‍ ‍mL NMS liquid medium at 30°C under an approximately 10% (v/v) methane gas atmosphere in a serum bottle (52×95‍ ‍mm, Mouth O.D. 20‍ ‍mm; DWK Life Science).

### Morphological characterization of MOB isolates

The colony morphology of MOB isolates was observed on NMS agar plates (1.5% [w/v]) and NMS gellan gum plates (0.4% [w/v]). Negatively stained cells for 2% (aqueous) uranyl acetate in MOB isolates were observed with a transmission electron microscope (H7500; Hitachi) at 100 kV and photographed using a CCD camera (Advanced Microscopy Technique) connected to the microscope. Intracytoplasmic structures were observed in ultra-thin sections of cells fixed with 2% (v/v) glutaraldehyde and 2% (w/v) osmium tetroxide and stained with 2% (w/v) uranyl acetate and lead stain solution with a JEM-1400Flash (JEOL) at 100 kV at the Hanaichi UltraStructure Research Institute.

### Phylogenetic ana­lysis of 16S rRNA and pmoA gene sequences of MOB isolates

Partial fragments of the 16S rRNA and *pmoA* (particulate methane monooxygenase [pMMO]) genes were amplified by colony PCR with EmeraldAmp PCR Master Mix (Takara Bio) and the primers 27f/1492r (Weisburg *et al.*, 1991) and A189f (Holmes *et al.*, 1995)/mb661r (Costello and Lidstrom, 1999). The sequencing ana­lysis was outsourced to Eurofins Genomics. Close relatives were searched on EzBioCloud (https://www.ezbiocloud.net/resources/16s_download) and BLAST (https://blast.ncbi.nlm.nih.gov/Blast.cgi). Phylogenetic trees were constructed using ClustalW by the neighbor-joining method with the Kimura two-parameter model and a bootstrap ana­lysis with 1,000 replications by MEGA11 (Stecher *et al.*, 2020; Tamura *et al.*, 2021).

### Extraction and purification of genomic DNA (gDNA) from MOB isolates

Wet cells (0.1–0.4 g) of MOB isolates and *Methylocystis echinoides* LMG 27198^T^ (=IMET 10491^T^), which was obtained from the BCCM/LMG (Ghent, Belgium), collected from NMS liquid medium were suspended in 500‍ ‍μL TESS buffer (25‍ ‍mM Tris-HCl [pH 7.4], 5‍ ‍mM EDTA, 50‍ ‍mM NaCl, and 25% [w/v] sucrose). Cells were lysed with 1% SDS, 0.2‍ ‍mg mL^–1^ lysozyme, and 1.4‍ ‍mg mL^–1^ proteinase K. Crude gDNA was repeatedly purified with a phenol–chloroform–isoamyl alcohol mixture (Merck KGaA) and chloroform–isoamyl alcohol mixture (Merck KGaA). RNA was digested by a treatment with 0.4‍ ‍mg mL^–1^ RNase A at 37°C for 60‍ ‍min. The concentration and purity of gDNA were measured using Nanodrop and Qubit (Thermo Fisher Scientific).

### Whole genome ana­lysis of MOB isolates

Genome sequencing was performed using PacBio Sequel II (Pacific Biosciences of California) or PacBio Revio sequencer (Pacific Biosciences of California). DNA libraries were constructed with SMRTbell Express Template Prep Kit v2.0 (Pacific Biosciences of California) with a cut-off at 10–50‍ ‍kb and/or 15–50‍ ‍kb using the BluePippin size selection system (Sage Science). The assembly was conducted using SMRTLink v10.2 (Pacific Biosciences of California), Canu ver2.0 (Koren *et al.*, 2017), SMRTLink v13 (Pacific Biosciences of California), or SMRTLink v12 (Pacific Biosciences of California). The circularity of contigs was confirmed by Circlator 1.5.5 (Hunt *et al.*, 2015). In a further quality check, error-corrected reads were aligned to contigs and gaps were assessed as described by Masuda *et al.* (2024).

### Comparative genome ana­lysis

Average nucleotide identity (ANI) and digital DNA-DNA hybridization (dDDH) values between isolated MOB and type strains or related MOB strains were calculated with the ANI calculator (https://www.ezbiocloud.net/tools/ani) (Yoon *et al.*, 2017) and Genome–to–Genome Distance Calculator 3.0 (https://ggdc.dsmz.de/ggdc.php) (Meier-Kolthoff *et al.*, 2022). Prokka version 1.13 (Seemann, 2014) was employed for genome annotation. The construction of the phylogenetic tree of whole genomes was performed by core genome identification using PIRATE version 1.0.5 (Bayliss *et al.*, 2019). Subsequent phylogenetic inference conducted with SeaView version 5.0.4 (Gouy *et al.*, 2010) was performed using the distance-based BioNJ algorithm with the Kimura two-parameter model and 1,000 bootstrap replications.

### Identification of genes for methane oxidation and nitrogen fixation

Gene clusters of methane monooxygenase genes (*pmoCAB1*), a *pmoCAB*-like gene (*pmoCAB2*), sequence-divergent particulate monooxygenase genes (*pxmABC*), soluble methane monooxygenase genes (*mmoZXY*), Ca-dependent methanol dehydrogenase genes (*mxaFJG*), and nitrogenase genes (*nifHDK*) for MOB isolates in the present study were extracted from genomes based on annotation by Prokka. BlastKOALA version 3.1 (Kanehisa *et al.*, 2016) was used to annotate the La/Ce-dependent methanol dehydrogenase gene (*xoxF*). The *mmoX* and *mxaF* gene sequences of MOB isolates in previous studies were collected from the NCBI database (https://www.ncbi.nlm.nih.gov/nucleotide/) for the construction of phylogenetic trees. The *mmoX* genes in *Methylomonas* spp., *Methylocystis* spp., and *Methylosinus* spp. were subjected to the ana­lysis after checking the operon of *mmoXYZ*. Phylogenetic trees of the *mmoX* and *mxaF* genes were constructed using the same methods for the 16S rRNA and *pmoA* gene sequences.

### Accession numbers

The accession numbers of the 16S rRNA gene, pmoA gene, genome sequences, and strains in this study were as follows: LC760236–LC760239, LC842179–LC842198, AP038927–AP038975, JCM 36253–JCM 36256, JCM 37584–JCM 37591, NBRC 116389–NBRC 116394 and KCTC 8600–KCTC8603.

## Results

### Isolation of MOB from rice roots

The present study used rice roots originating from three rice varieties as inoculant materials to isolate MOB. One hundred MOB cultures were successfully obtained from all the rice roots. Not only the enrichment culture method, but also the direct spread plate method was applicable to obtain MOB cultures from rice roots; however, the number of cultures obtained by the direct spread plate method was lower than that by the enrichment culture method (Table S1). In all cases, repeated and careful purification steps were needed to purify MOB strains (Table S2) because contaminating bacteria, *e.g.*, methylotrophs, were present in the MOB colonies that formed on the plates. Among growing MOB on plates, faster colonies formed after approximately 5 days and slower ones over a 2-week incubation. Although methane consumption varied depending on the shape of the culture vessel, when seeded in serum bottles and shaken and incubated at 30°C, changes in turbidity were visible within 5 days at the latest. Methane consumption was observed within 24‍ ‍h for all isolates when the strains were grown in test tubes with 10‍ ‍mL NMS liquid medium at 30°C under 30% methane and shaking. In the present study, 12 MOB isolates were successfully purified.

### Phylogenetic characteristics of MOB isolates

**Fig. 1.**
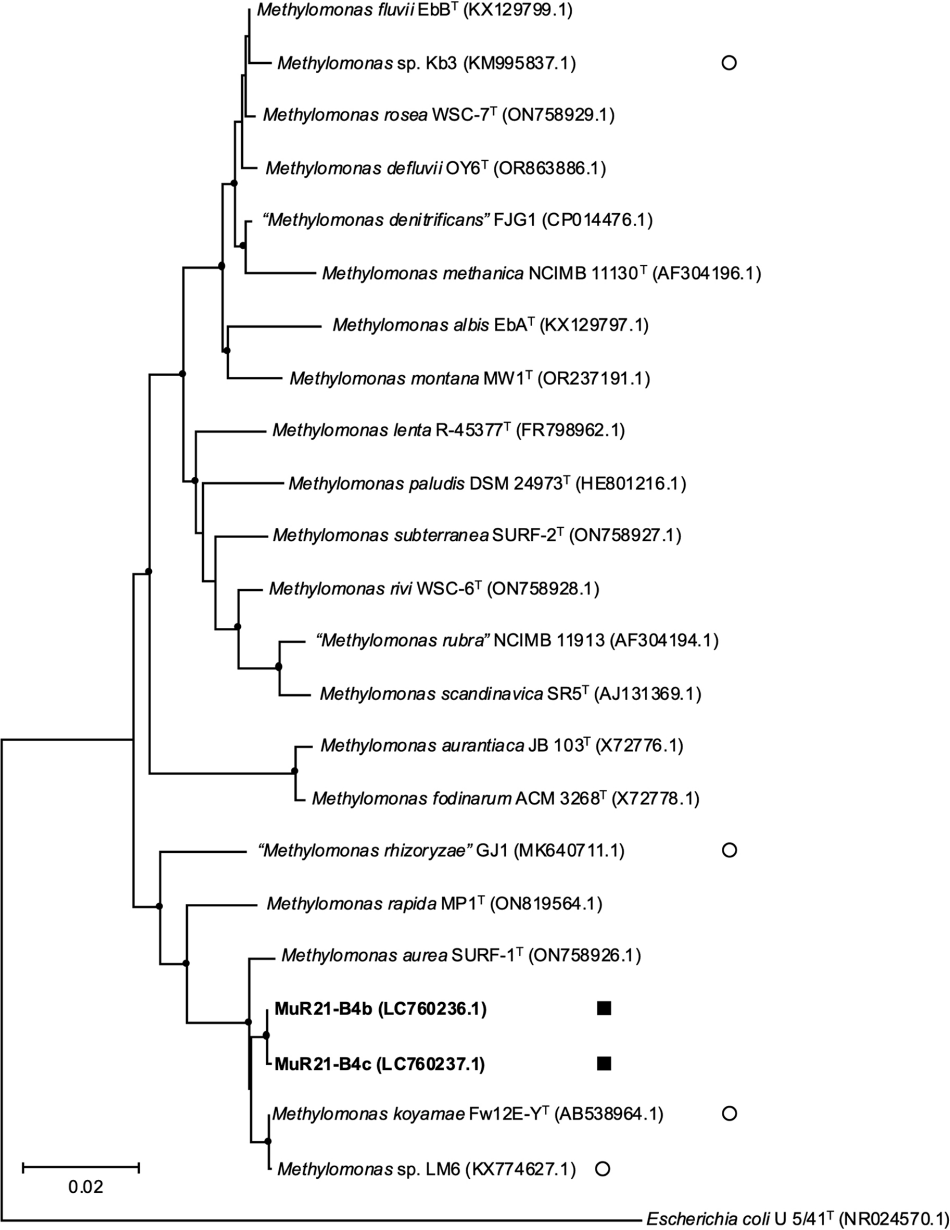
Neighbor-joining phylogenetic tree based on 16S rRNA gene sequences of *Methylomonas* sp. strains. Closed square, isolates from rice roots in this study; open circle, isolates from rice fields other than rice roots. Bar, 0.02 substitutions per nucleotide sequence position. Filled circles indicate internal nodes with at least 50% bootstrap support from 1,000 data resampling. The tree was rooted using *Escherichia coli* U 5/41^T^ as the outgroup. GenBank accession numbers are given in parentheses.

**Fig. 2.**
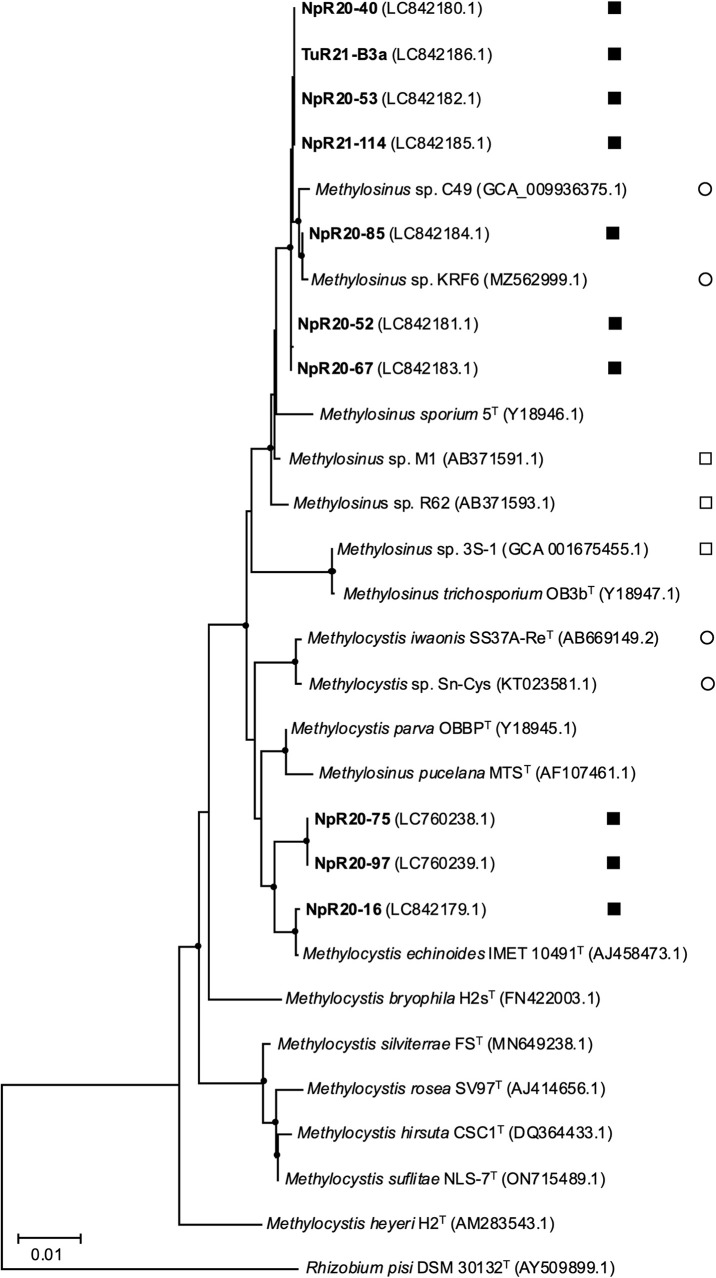
Neighbor-joining phylogenetic tree based on 16S rRNA gene sequences of type II MOB strains. Closed square, isolates from rice roots in this study; open square, isolates from rice roots in previous studies; open circle, isolates from rice fields other than rice roots. Bar, 0.01 substitutions per nucleotide sequence position. Filled circles indicate internal nodes with at least 50% bootstrap support from 1,000 data resampling. The tree was rooted using *Rhizobium pisi* DSM 30132^T^ as the outgroup. GenBank accession numbers are given in parentheses.

A phylogenetic ana­lysis of the 16S rRNA and *pmoA* gene sequences showed that two strains belonged to type I MOB and ten strains to type II MOB (Fig. 1, 2, and S1). The MuR21-B4b and MuR21-B4c strains in type I MOB were closely related to *Methylomonas koyamae* Fw12E-Y^T^ with similarity values >99.4% for the 16S rRNA gene and to *Methylomonas aurea* SURF-1^T^ with similarity values >95.1% for the *pmoA* gene. Among type II MOB strains, three strains (NpR20-16, NpR20-75, and NpR20-97) were related to *Methylocystis echinoides* IMET10491^T^ with similarity values >99.1% for the 16S rRNA gene and 95.8% for the *pmoA* gene. The seven other strains (NpR20-40, NpR20-52, NpR20-53, NpR20-67, NpR20-85, NpR21-114, and TuR21-B3a) were related to *Methylosinus sporium* 5^T^ with similarity values >99.0% for the 16S rRNA gene and 97.8% for the *pmoA* gene.

The phylogenetic trees shown in Fig. 1, 2, and S1 included MOB isolates from rice roots and other sources in the rice field in previous studies. Five strains in the genera *Methylomonas* (MuR21-B4b and MuR21-B4c) and *Methylocystis* (NpR20-16, NpR20-75, and NpR20-97) were located apart from the MOB isolates from rice fields in the trees. The seven strains (NpR20-40, NpR20-52, NpR20-53, NpR20-67, NpR20-85, NpR21-114, and TuR21-B3a) in *Methylosinus* belonged to the same clade as the MOB isolates from rice roots in a previous study (Takeda *et al.*, 2008), but were more closely related to the MOB isolates from rice field soil (Yasuda *et al.*, 2020b; Rahalkar *et al.*, 2021).

### Morphological and physiological characteristics of MOB isolates

**Fig. 3.**
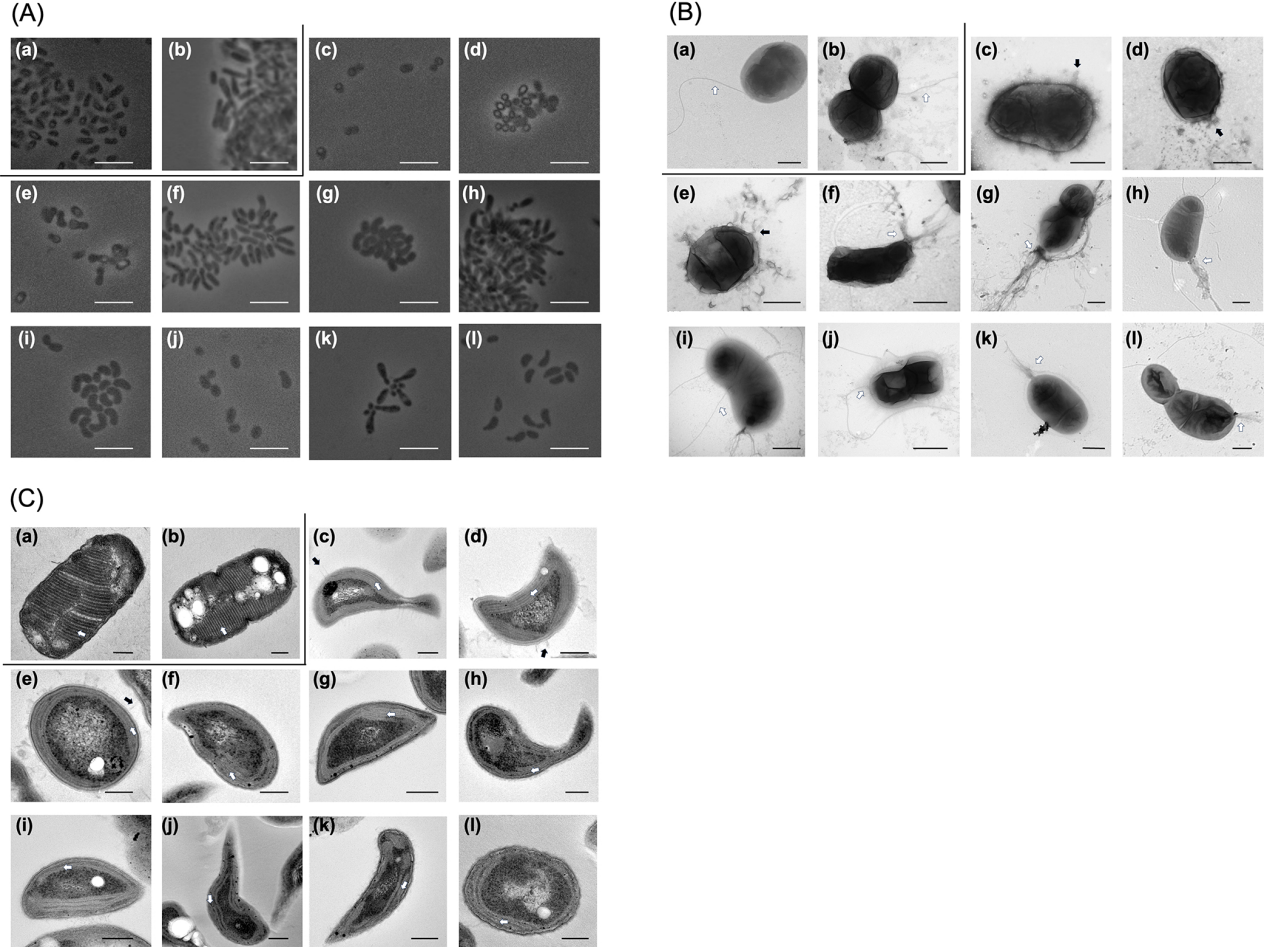
(A) Phase-contrast light micrographs, (B) negative stain transmission electron micrograph, and (C) transmission electron micrograph of ultrathin sections of MOB strains isolated from rice roots. Strains: (a), MuR21-B4b; (b), MuR21-B4c; (c), NpR20-16; (d), NpR20-75; (e), NpR20-97; (f), NpR20-40; (g), NpR20-52; (h), NpR20-53; (i), NpR20-67; (j), NpR20-85; (k), NpR21-114; (l), TuR21-B3a. (a) and (b), (c) to (e), and (f) to (l) were related to *Methylomonas* sp., *Methylocystis* sp., and *Methylosinus* sp., respectively. Bars represent 5‍ ‍μm (A), 0.5‍ ‍μm (B), and 0.2‍ ‍μm (C). The black arrow represents a tubular structure. The white arrows indicate a flagellum (B) and intracytoplasmic membrane (ICM) (C).

The cell morphologies and intracytoplasmic structures of MOB isolates are shown in Fig. 3, and the morphological and physiological characteristics of the isolates and closely related type strains are summarized in Table 1. The MuR21-B4b and MuR21-B4c strains, related to *Methylomonas*, were rod shapes with dimensions of 0.8–1.9×1.1–2.2‍ ‍μm and were motile with a polar flagellum. They formed a pellicle on the surface of the liquid culture. Rosettes were sometimes observed in liquid cultures. These strains did not form colonies on solid medium plates. Strains NpR20-16, NpR20-75, and NpR20-97, which were related to *Methylocystis*, were rod shapes with dimensions of 0.7–1.4×0.9–2.2‍ ‍μm and were not motile. All three strains had tubular structures on the cell surface (Fig. 3B c, d, and e) and formed cysts. Rosettes were observed in the cultures of NpR20-75 and NpR20-97, but not in that of NpR20-16. The colony morphology of strain NpR20-16 was circular, convex, entire, and white on NMS agar plates. The NpR20-75 and NpR20-97 strains did not form colonies on NMS agar plates, but formed circular, convex, entire, and pale pink colonies on NMS gellan gum plates. The NpR20-40, NpR20-52, NpR20-53, NpR20-67, NpR20-85, NpR21-114, and TuR21-B3a strains, related to *Methylosinus*, were rod, kidney, or pear shapes with dimensions of 0.5–1.7×0.8–3.5‍ ‍μm, were motile, and formed rosettes. The colonies that formed on NMS agar plates were circular, convex, entire, and white. The NpR20-40, NpR20-52, NpR20-53, NpR21-114, and TuR21-B3a strains had a polar flagellar tuft, while the NpR20-67 and NpR20-85 strains possessed peritrichous flagella and a polar flagellum, respectively.

The MuR21-B4b and MuR21-B4c strains showed the typical internal cytoplasmic membrane (ICM) structure of type I MOB, while the NpR20-16, NpR20-75, NpR20-97, NpR20-40, NpR20-52, NpR20-53, NpR21-114, and TuR21-B3a strains had the typical ICM of type II MOB (Fig. 3C).

### Genomic characteristics of MOB isolates

Genome sequences were elucidated by various tools (Table S3) and genomic characteristics are shown in Table 2. A complete genome at the assembly level was obtained for all isolates and *Methylocystis echinoides* LMG 27198^T^. The genome sizes of the isolates and G+C contents ranged from 4.48 to 4.99‍ ‍Mbp with 4,147–4,640 CDSs and from 55.6 to 64.8%, respectively. All the isolates harbored one or more plasmids with 0.12–0.22‍ ‍Mbp; the MuR21-B4b, MuR21-B4c, NpR20-16, NpR20-75, and NpR20-97 strains contained only one plasmid (Table 2a and b), while the NpR20-40, NpR20-52, NpR20-53, NpR20-67, NpR20-85, NpR21-114, and TuR21-B3a strains possessed between two and five plasmids (Table 2c).

Genes associated with methane monooxygenase, methanol dehydrogenase, and nitrogenase are shown in Table 2. All the isolates had one to two *pmoCAB1* and one *mxaFJG*, while *pmoCAB2* and *pxmABC* were only found in the strains related to *Methylosinus* and *Methylocystis*. *mmoZXY* was found in all the isolates in *Methylosinus*, but was not present in MuR21-B4b or MuR21-B4c in *Methylomonas* or in NpR20-16, NpR20-75, or NpR20-97 in *Methylocystis*. *xoxF* was found in all the isolates; however, its number differed. The number of the gene in the strains related to *Methylomonas* and *Methylosinus* was one and two, while that in the strains related to *Methylocystis* was four to five. All the isolates had one* nifHDK*. Phylogenetic trees based on the *mmoX* and *mxaF* gene sequences of the MOB strains are shown in Fig. S2 and S3.

### Genomic relatedness of MOB isolates

The similarity values of ANI and dDDH between the isolates and related MOB strains are shown in Table 3. The MuR21-B4b and MuR21-B4c strains were the closest to the type I MOB, *Methylomonas aurea* SURF-1^T^ with ANI and dDDH values of 89 and 37%, respectively (Table 3a). Among the ten isolates belonging to type II MOB, the NpR20-16, NpR20-75, and NpR20-97 strains were closely related to *Methylocystis echinoides* IMET10491^T^. Strain NpR20-16 was closely related to *Methylocystis echinoides* LMG 27198^T^ with ANI and dDDH values of 97 and 74%, respectively (Table 3b), indicating that the strain belonged to the species *Methylocystis echinoides*. However, the genome size and number of plasmids differed between strain NpR20-16 and *Methylocystis echinoides* LMG 27198^T^. The NpR20-75 and NpR20-97 strains showed ANI and dDDH values of 85 and 28%, respectively, for *Methylocystis echinoides* LMG 27198^T^. The seven other strains, NpR20-40, NpR20-52, NpR20-53, NpR20-67, NpR20-85, NpR21-114, and TuR21-B3a, exhibited close relationships with *Methylosinus sporium* 5^T^ with ANI and dDDH values of 97–98 and 73–78%, respectively (Table 3c). Phylogenomic trees based on genome sequences confirmed their phylogenetic relatedness; however, differences in distances were observed even among isolates closely related to *Methylosinus sporium* 5^T^ (Fig. S4).

## Discussion

### Phylogenetic diversity of MOB isolates

Previous studies showed that type II MOB were predominant in the rice roots of *Oryza sativa* L. subsp. *japonica* cv. Nipponbare grown in the same experimental plot without nitrogen fertilizer (Ikeda *et al.*, 2014; Hara *et al.*, 2022), which is consistent with the present results. Ikeda *et al.* (2014) revealed that type II MOB were preferentially present in the rice roots of Nipponbare based on the phylogenetic compositions of 16S rRNA gene clone libraries. Hara *et al.* (2022) indicated that type II MOB were present in approximately 7% of rice roots of Nipponbare using an amplicon sequence ana­lysis of the 16S rRNA gene and FISH observations. In addition, three type II MOB strains were previously isolated from the rice roots of *Oryza sativa* L. subsp. *japonica* cv. Mutsuhomare, Yumeakari, and Nipponbare (Takeda *et al.*, 2008; Bao *et al.*, 2016). These findings indicate that the major MOB in/on the rice roots of Nipponbare were type II MOB, which may also be the case in other rice cultivars’ roots. We utilized rice roots as the isolation source, obtained 100 MOB (Table S1), and ultimately gained 12 MOB strains: two *Methylomonas* sp. (type I), three *Methylocystis* sp. (type II), and seven *Methylosinus* sp. (type II) (Table 1 and Fig. 1, 2, and S1). Only three strains of MOB had previously been isolated from rice roots and all of them belonged to *Methylosinus* (Takeda *et al.*, 2008; Bao *et al.*, 2016). This is the first study to isolate the genus *Methylomonas* in type I MOB and the genus *Methylocystis* in type II MOB from rice roots.

Beyond rice roots, several MOB isolates from rice fields based on the 16S rRNA and *pmoA* genes have been reported: *Methylogaea oryzae* (Geymonat *et al.*, 2011), *Methylomonas koyamae* (Ogiso *et al.*, 2012; Lee *et al.*, 2020), *Methylomagnum ishizawai* (Khalifa *et al.*, 2015; Frindte *et al.*, 2017), *Methyloterricola oryzae* (Frindte *et al.*, 2017), *Methylocucumis oryzae* (Pandit and Rahalkar, 2019; Mohite *et al.*, 2023), *Methylocystis iwaonis* (Kaise *et al.*, 2023), *Methylococcus mesophilus* (Awala *et al.*, 2023), “*Methylotetracoccus oryzae*” (Ghashghavi *et al.*, 2019), “*Methylomonas rhizoryzae*” (Zhu *et al.*, 2020), “*Candidates* Methylobacter oryzae” (Rahalkar *et al.*, 2019), *Methylomonas* sp. (Rahalkar and Pandit, 2018), *Methylomicrobium* sp. (Rahalkar *et al.*, 2021), *Methylocystis* sp. (Pandit *et al.*, 2016), and *Methylosinus* sp. (Dianou and Adachi, 1999; Yasuda *et al.*, 2020b; Mohite *et al.*, 2023). However, the present study showed that MOB strains also differed from the isolates in previous studies based on the phylogenetic characteristics of the 16S rRNA and *pmoA* genes, as shown in Fig. 1, 2, and S1. In addition, the complete genome sequences of 12 MOB strains from rice roots elucidated in the present study revealed the distinct characteristics of the strains in the general features of their genomes (Table 2) and phylogenomic relationships with other MOB strains (Table 3 and Fig. S4). Genomic information on MOB isolated from rice roots is currently available for only one strain (Bao *et al.*, 2016). Given these results, the eco-collection of MOB from rice roots and rice fields has been largely expanded.

### Employing several culture methods may be effective to obtain diverse MOB from the rice field ecosystem

Type I and II MOB were both isolated from rice root samples in 2021, while all the strains from rice root samples in 2020 were type II MOB (Table 1). This difference in the types of MOB obtained between the samples in 2020 and 2021 may be attributed to the storage temperature conditions of rice root samples: –30°C in 2020 and 4°C in 2021, because type I MOB generally have no tolerance to freeze-thaw (Nesterov *et al.*, 1986). Conversely, using frozen samples as inoculants for enrichment cultures may be advantageous for the selective screening of type II MOB.

To clarify why we obtained various MOB isolates from rice roots, we compared the present study with similar studies on the cultivation, isolation, and characterization of MOB from a Japanese rice paddy field (Dianou *et al.*, 2012; Ogiso *et al.*, 2012; Khalifa *et al.*, 2015; Kaise *et al.*, 2023). In this series of studies, seven compartments of the rice paddy field, including rice roots, were used as inoculation sources, 13 MOB cultures were obtained, and three novel MOB strains, *Methylomonas koyamae* Fw12E-Y^T^, *Methylomagnum ishizawai* RS11D-Pr^T^, and *Methylocystis iwaonis* SS37A-Re^T^, were ultimately obtained. They used two isolation techniques (colony isolation and dilution-to-extinction methods) and four culture media (NMS agar, 1a liquid and agar [Leadbetter and Foster, 1958], and ammonium mineral medium agar [Bosse and Frenzel, 1997]). In contrast, we only employed rice roots as the isolation source, but exploited two conditions for cultivation, four isolation techniques, and four culture media for isolation, resulting in the isolation of 12 MOB strains. It may have been effective to employ various isolation techniques to obtain a larger number of various MOB strains even though only rice roots were used. In addition, the following two factors may be important: performing dilution-to-extinction with the broth at the initial growth stage and using gellan gum as a coagulant. Using the broth at the initial growth stage may have avoided the growth of heterotrophs that consume the metabolites of MOB. Agar contains trace amounts of furan-2-carboxylic acids, which inhibit the multiplication of some slow-growing bacteria on plates (Hara *et al.*, 2012). The NpR20-75 and NpR20-97 strains were grown on NMS gellan gum plates, but not on NMS agar plates (Table 1b). Therefore, gellan gum represents a good alternative to agar for the isolation of various MOB.

### Classification of MOB isolates by morphological, physiological, and genetic features

Morphological and physiological characterizations were conducted for MOB strains (Fig. 3 and Table 1). The MuR21-B4b and MuR21-B4c strains showed distinct features, such as not growing on NMS agar plates, from the type strain *Methylomonas koyamae* Fw12E-Y^T^ (Ogiso *et al.*, 2012) of closely related species, which was isolated from the floodwater of a rice field. The MOB strains NpR20-16, NpR20-75, and NpR20-97, which were closely related to the species *Methylocystis echinoides*, also exhibited several distinct characteristics from the type strain *Methylocystis echinoides* IMET 10491^T^ (Gal’chenko *et al.*, 1978) of the species: the cell sizes of the strains were larger than that of *Methylocystis echinoides* LMG 27198 ^T^; strain NpR20-16 formed colonies on agar medium; strains NpR20-75 and NpR20-97 grew on gellan gum medium, but not on agar medium, and formed rosettes in liquid medium. The non-motile trait and presence of tubular cell structures were common among the strains. The strains related to the genus *Methylosinus*, NpR20-40, NpR20-52, NpR20-53, NpR20-67, NpR20-85, NpR21-114, and TuR21-B3a, were commonly motile, while flagellation differed among the strains. Five strains, except for NpR20-67 and NpR20-85, had polar flagellar tufts. In contrast, the NpR20-67 and NpR20-85 strains had peritrichous flagella and a polar flagellum, respectively, a distinct feature from that found in the closely related species *Methylosinus sporium* 5^T^ (Whittenbury *et al.*, 1970). The phenotypic characterization of the MOB strains from rice roots, for which further investigations are warranted, revealed the distinctiveness of the strains from the other MOB isolates from rice fields.

According to genome relatedness indexes between MOB isolates and species of the genera *Methylomonas* and *Methylocystis* (Table 3a and 3b), as well as the number of CDS (Table 2a and 2b), the MuR21-B4b, MuR21-B4c, NpR20-75, and NpR20-97 strains represent a novel species in the genus *Methylomonas* or *Methylocystis*. ANI and dDDH values indicate that the NpR20-40, NpR20-52, NpR20-53, NpR20-67, NpR20-85, NpR21-114, and TuR21-B3a strains belonged to *Methylosinus sporium* (Table 3c). However, genomic relatedness varied between the seven strains and differences were observed in the genome size and numbers of CDS and plasmids (Table 2c and 3c). In addition, strain NpR20-85 exhibited the unique phenotypic feature of flagellation with a polar flagellum, which was different from that of *Methylosinus sporium* 5^T^ (Table 1c). Therefore, the MOB strains related to *Methylosinus sporium* may represent a novel subspecies.

The diversity of the genes related to methane oxidation, methanol dehydration, and nitrogen fixation in MOB isolates is shown in Table 2 and Fig. S1–S3. Regarding methane oxidation, *pxmABC* genes were reported in gammaproteobacterial MOB in the genera *Methylomonas*, *Methylobacter*, and *Methylomicrobium* (Tavormina *et al.*, 2011). *pxmABC* genes were not present in the MuR21-B4b and MuR21-B4c strains, representing putative novel species within the genus *Methylomonas*, but were found in the NpR20-75 and NpR20-97 strains, representing putative novel species within the genus *Methylocystis*. This variation in the existence of *pxmABC* genes may indicate that MOB have a divergent strategy for methane oxidation according to the physiological traits of the respective MOB isolates. *nifHDK* genes are necessary for nitrogen fixation and the majority of type II MOB have this gene cluster (Hara *et al.*, 2022). Therefore, type II MOB may contribute not only to reducing methane emissions from rice fields, but also to supplying nitrogen to rice plants (Minamisawa, 2022). All of the MOB isolates, not only in type II, but also in type I, in the present study possessed the *nifHDK* gene cluster. This may be attributed in part to the MOB strains being isolated from the roots of rice plants grown long term at a paddy field plot without nitrogen fertilizer, as previously reported by Hara *et al.* (2022).

The application of MOB has recently been used in various fields, *e.g.*, microbial inoculants for reducing methane emissions from rice fields (Davamani *et al.*, 2020; Minamisawa, 2022). Further efforts to isolate cultivable MOB strains are still needed and are very important for expanding the eco-collection of MOB by fulfilling bacterial resources with well-characterized strains for these application trials of MOB.

### Agenda for the future

The present study revealed some phenotypic and genotypic characteristics of MOB strains isolated from rice roots. Some of these strains may represent novel species and the others were affiliated with known species; however, some distinctiveness was observed in morphological, physiological, and genomic features among the strains. Further investigations of the phenotypes of the strains, such as physiological and chemotaxonomic characteristics, are needed for the identification of strains and proposals of novel species. The present results indicate the importance of a genome ana­lysis as well as morphological, physiological, and phylogenetical ana­lyses of isolated MOB strains for revealing the diversity of cultivable MOB inhabitants in rice roots. We are planning to conduct comparative genomics of the isolated strains with other MOB strains, which will provide important information for understanding the eco-physiology of MOB inhabitants. Genomic information together with the phenotypic features of strains will be useful for their future application to the mitigation of methane emissions from rice fields. In addition, further efforts to isolate not yet cultivated MOB strains from rice roots and other compartments in rice fields are still required and are very important for expanding the eco-collection of MOB by fulfilling bacterial resources with well-characterized strains for the future application of the MOB strains.

## Conclusion

**Fig. 4.**
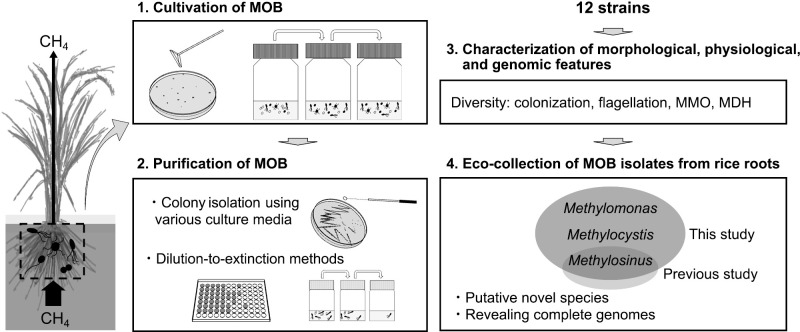
Graphical summary of this study.

We aimed to gain cultivable MOB isolates and expand the collection of MOB inhabiting rice fields. Twelve strains consisting of two type I and ten type II MOB were obtained from rice roots and characterized by whole genome sequencing as well as morphological, physiological, and phylogenetical ana­lyses (Fig. 4). The results obtained herein show that the strains exhibited some distinctiveness in their genotypic and phenotypic features; two strains of type I MOB and two strains of type II MOB represent potentially novel species. The present results indicate the importance of a genome ana­lysis as well as morphological, physiological, and phylogenetic ana­lyses of isolated MOB strains for revealing the diversity of cultivable MOB inhabitants in rice roots. In addition, this study indicates that rice roots still have potential as an important isolation source of cultivable MOB, and we will be able to further expand the eco-collection. Therefore, we need to continue our efforts to isolate not yet cultivated MOB strains from their habitats in rice fields.

## Citation

Oe, F., Shinjo, R., Masuda, S., Shibata, A., Shirasu, K., Hashimoto, S., et al. (2025) Expanding the Eco-collection of Methane-oxidizing Bacteria Inhabiting Rice Roots: Cultivation, Isolation, and Genomic Characterization of Isolates. *Microbes Environ ***40**: ME25012.

https://doi.org/10.1264/jsme2.ME25012

## Supplementary Material

Supplementary Material

## Figures and Tables

**Table 1.** Characteristics of MOB strains isolated from paddy fields related to (a) *Methylomonas* sp., (b) *Methylocystis* sp., and (c) *Methylosinus* sp.

(a) *Methylomonas* sp.			
Strain	MuR21-B4b	MuR21-B4c	*Ref.*1
Morphology	rod	rod	rod
Cell width (μm)	0.8–1.9	0.9–1.4	0.8–1.1
Cell length (μm)	1.1–2.2	1.2–2.1	1.2–2.5
Motility	+	+	+
Flagellum	polar flagella	polar flagella	polar flagella
Colony formation	–	–	+
Colony morphology	–	–	round
Colony color	–	–	pink, orange
MeOH utilization	+	+	+
Surface pellicle	+	+	–
Rosette formation	+	+	–
Isolation source	Muha, rice roots, 2021	Muha, rice roots, 2021	floodwater of a paddy field

(b) *Methylocystis* sp.				
Strain	NpR20-16	NpR20-75	NpR20-97	*Ref.*2
Morphology	rod	rod	rod	reniform, coccobacilli, rods
Cell width (μm)	0.8–1.4	0.7–1.0	0.8–1.3	0.6
Cell length (μm)	1.3–2.2	0.9–1.6	1.1–1.8	0.8–1.2
Motility	–	–	–	–
Flagellum	–	–	–	–
Colony formation	+	+*	+*	+
Colony morphology	circular/convex/entire	circular/convex/entire	circular/convex/entire	circular/convex/entire
Colony color	white	white	pale pink	white/pale pink
MeOH utilization	+	+	+	+
Surface pellicle	–	–	–	–
Rosette formation	–	+	+	–
Cyst formation	+	+	+	–
Isolation source	Nipponbare, rice roots, 2020	Nipponbare, rice roots, 2020	Nipponbare, rice roots, 2020	sewage sludge

(c)* Methylosinus* sp.								
Strain	NpR20-40	NpR20-52	NpR20-53	NpR20-67	NpR20-85	NpR21-114	TuR21-B3a	*Ref.*3
Morphology	rod, kidney	rod, pear	rod, pear	rod, pear	rod	rod	rod, pear	rod, vibrioid
Cell width (μm)	0.9–1.3	0.9–1.5	0.7–1.3	0.8–1.4	1.0–1.6	0.8–1.7	0.5–1.3	0.5–1.0
Cell length (μm)	1.1–2.3	1.3–2.9	1.3–2.7	1.2–2.3	1.6–3.1	1.1–3.0	0.8–3.5	1.5–3.0
Motility	+	+	+	+	+	+	+	+
Flagellum	polar flagellar tuft	polar flagellar tuft	polar flagellar tuft	peritrichous flagella	polar flagella	polar flagellar tuft	polar flagellar tuft	polar flagellar tuft
Colony formation	+	+	+	+	+	+	+	+
Colony morphology	circular/convex/entire	circular/convex/entire	circular/convex/entire	circular/convex/entire	circular/convex/entire	circular/convex/entire	circular/convex/entire	circular/convex/entire
Colony color	white	white	white	white	white	white	white	brown
MeOH utilization	+	+	+	+	+	+	+	+
Surface pellicle	–	–	–	–	–	–	–	–
Rosette formation	+	+	+	+	+	+	+	+
Cyst formation	–	–	–	–	–	–	–	–
Isolation source	Nipponbare, rice roots, 2020	Nipponbare, rice roots, 2020	Nipponbare, rice roots, 2020	Nipponbare, rice roots, 2020	Nipponbare, rice roots, 2020	Nipponbare, rice roots, 2021	Tupa 121-3, rice roots, 2021	groundwater aquifer

*Ref.*1, *Methylomonas koyamae* Fw12E-Y^T^ (Ogiso *et al.*, 2012); *Ref.*2, *Methylocystis echinoides* IMET 10491^T^ (Gal’chenko *et al.*, 1978); *Ref*.3, *Methylosinus sporium* 5^T^ (Whittenbury *et al.*, 1970). +, positive; –, negative. +* indicates colony formation on NMS gellan gum plates.

**Table 2.** Characteristics of whole genomes of MOB isolated from rice roots related to (a) *Methylomonas* sp., (b) *Methylocystis* sp., and (c) *Methylosinus* sp..

(a) *Methylomonas* sp.				
Strain	MuR21-B4b	MuR21-B4c	*Ref.*1	*Ref.*2
Genome size (Mbp)	4.63	4.62	4.81	5.10
CDS	4130	4147	4226	4481
G+C percent	55.6	55.6	56.0	56.1
Level	Complete	Complete	Contig	Complete
No. of contigs/scaffolds	2	2	93	2
Plasmid	1	1	–	1
	(0.22‍ ‍Mbp)	(0.22‍ ‍Mbp)	–	(0.24‍ ‍Mb)
tRNA	51	51	43	51
rRNA (5S, 16S, 23S)	3, 3, 3	3, 3, 3	2, 1, 1	3, 3, 3
Methane monooxygenase genes				
*pmoCAB1*	1	1	1	1
*pmoCAB2*	0	0	0	0
*pxmABC*	0	0	1	1
*mmoXYZ*	0	0	0	0
Methanol dehydrogenase genes				
*mxaFJG*	1	1	1	1
*xoxF*	1	1	1	1
Nitrogenase genes				
nifHDK	1	1	1	1

(b) *Methylocystis* sp.				
Strain	NpR20-16	NpR20-75	NpR20-97	*Ref.*3
Genome size (Mbp)	4.58	4.50	4.50	5.34
CDS	4412	4205	4196	5130
G+C percent	64.5	62.9	62.9	63.8
Level	Complete	Complete	Complete	Complete
No. of contigs/scaffolds	2	2	2	6
Plasmid	1	1	1	5
	(0.19‍ ‍Mbp)	(0.12‍ ‍Mbp)	(0.12‍ ‍Mbp)	(0.09–0.48‍ ‍Mbp)
tRNA	56	53	53	53
rRNA (5S, 16S, 23S)	3, 3, 3	3, 3, 3	3, 3, 3	3, 3, 3
Methane monooxygenase genes				
*pmoCAB1*	2	2	2	2
*pmoCAB2*	0	0	0	0
*pxmABC*	0	1	1	2
*mmoXYZ*	0	0	0	0
Methanol dehydrogenase genes				
*mxaFJG*	1	1	1	1
*xoxF*	5	4	4	5
Nitrogenase genes				
*nifHDK*	1	1	1	1

(c) *Methylosinus* sp.								
Strain	NpR20-40	NpR20-52	NpR20-53	NpR20-67	NpR20-85	NpR21-114	TuR21-B3a	*Ref.*4
Genome size (Mbp)	4.93	4.68	4.56	4.48	4.99	4.86	4.50	4.44
CDS	4,640	4,350	4,329	4,205	4,587	4,537	4,165	4,049
G+C percent	64.4	64.6	64.8	64.7	64.5	64.4	64.7	64.8
Level	Complete	Complete	Complete	Complete	Complete	Complete	Complete	Contig
No. of contigs/scaffolds	6	5	5	4	6	5	3	114
Plasmid	5	4	4	3	5	4	2	–
	(0.08–0.19‍ ‍Mbp)	(0.05–0.20‍ ‍Mbp)	(0.02–0.22‍ ‍Mbp)	(0.05–0.20‍ ‍Mbp)	(0.02–0.30‍ ‍Mbp)	(0.08–0.19‍ ‍Mbp)	(0.14, 0.22‍ ‍Mbp)	–
tRNA	53	54	53	54	57	54	53	52
rRNA (5S, 16S, 23S)	2, 2, 2	2, 2, 2	2, 2, 2	2, 2, 2	2, 2, 2	2, 2, 2	2, 2, 2	1, 1, 1
Methane monooxygenase genes								
*pmoCAB1*	2	2	2	2	2	2	2	1
*pmoCAB2*	1	1	1	1	1	1	1	1
*pxmABC*	0	0	0	0	0	0	0	0
*mmoXYZ*	2	2	2	2	2	2	2	2
Methanol dehydrogenase genes								
*mxaFJG*	1	1	1	1	1	1	1	1
*xoxF*	2	2	2	2	2	2	2	2
Nitrogenase genes								
*nifHDK*	1	1	1	1	1	1	1	1

*Ref.*1, *Methylomonas aurea* SURF-1^T^ (Abin *et al.*, 2024); *Ref.*2, *Methylomonas koyamae* Fw12E-Y^T^ (Ogiso *et al.*, 2012); *Ref.*3, *Methylocystis echinoides* LMG 27198^T^ (Gal’chenko *et al.*, 1978; this study); *Ref.*4, *Methylosinus sporium* 5^T^ (Whittenbury *et al.*, 1970).

**Table 3.** Genome relatedness indexes between MOB isolates and species of genera (a) *Methylomonas*, (b) *Methylocystis*, and (c) *Methylosinus*. ANI and dDDH values are shown at the upper right and lower left of the diagonal, respectively. Accession numbers are presented in parentheses.

(a) *Methylomonas*																	
Strain		1	2	3	4	5	7	8	9	10	11	12	13	14	15	16	17
1	MuR21-B4b (AP038933, AP038934)		100	76.5	89.1	78.0	76.8	86.3	72.8	77.0	77.1	72.8	75.6	74.9	75.3	76.7	75.6
2	MuR21-B4c (AP038935, AP038936)	100		76.3	89.2	77.4	76.5	86.3	72.8	77.2	77.5	72.7	75.7	74.5	74.9	76.7	75.7
3	*Methylomonas albis* EbA^T^ (GCA_014850955.1)	20.5	20.5		76.4	85.5	86.1	76.7	73.6	86.4	81.5	73.1	75.3	73.2	75.1	85.5	74.8
4	*Methylomonas aurea* SURF-1^T^ (GCA_024505045.1)	37.1	37.1	20.2		78.6	74.8	88.1	72.7	77.3	77.7	72.6	76.0	74.7	75.7	76.7	76.3
5	*Methylomonas defluvii* OY6^T^ (GCA_033949435.1)	21.9	21.9	29.8	23.6		91.6	78.4	74.0	86.5	86.5	73.1	75.7	73.5	75.5	87.2	75.9
7	*Methylomonas fluvii* EbB^T^ (GCA_903064685.1)	20.7	20.7	31.1	21.0	44.5		77.6	77.6	86.5	81.3	73.0	76.0	73.4	75.8	87.2	75.1
8	*Methylomonas koyamae* Fw12E-Y^T^ (GCA_019669905.1)	31.1	31.1	21.0	34.3	23.0	21.9		73.0	77.3	77.7	72.6	76.3	74.7	75.5	76.9	75.7
9	*Methylomonas lenta* R-45370 (GCA_001644015.1)	19.5	19.5	19.8	19.2	20.5	20.6	19.5		73.7	74.1	72.5	75.3	72.5	76.3	73.6	75.4
10	*Methylomonas methanica* NCIMB 11130^T^ (GCA_001644045.1)	20.8	20.8	31.7	21.2	31.5	31.6	21.1	19.7		82.4	73.4	76.1	73.4	75.8	88.3	75.5
11	*Methylomonas montana* MW1^T^ (GCA_030490285.1)	20.8	20.8	24.7	21.2	24.6	24.8	21.2	20.1	25.7		73.3	76.4	74.1	76.1	81.7	76.0
12	*Methylomonas paludis* S2AM (GCA_018734325.1)	19.8	19.8	19.1	19.3	19.2	19.5	19.6	19.6	19.3	20.4		72.9	71.7	73.3	73.3	73.1
13	*Methylomonas rapida* MP1^T^ (GCA_024360925.2)	21.2	21.2	20.6	22.0	21.7	22.1	22.3	19.6	21.9	21.7	19.4		75.0	77.4	75.8	77.6
14	“*Methylomonas rhizoryzae*” GJ1 (GCA_008632455.1)	22.4	22.4	20.2	22.1	20.3	20.3	21.3	18.5	20.0	20.3	19.1	21.0		74.8	73.4	75.1
15	*Methylomonas rivi* WSC-6^T^ (GCA_024505165.1)	20.4	20.4	20.8	21.0	21.0	21.2	21.0	20.6	21.1	21.0	19.6	21.4	20.1		75.4	83.5
16	*Methylomonas rosea* WSC-7^T^ (GCA_024505055.1)	20.7	20.7	30.1	20.5	33.0	33.2	20.7	19.7	35.0	24.9	19.5	21.0	20.3	20.9		75.1
17	*Methylomonas subterranea* SURF-2^T^ (GCA_024505185.1)	21.1	21.1	20.2	22.9	21.2	20.6	20.7	19.9	20.8	21.3	19.5	21.5	21.3	27.4	20.6	

(b) *Methylocystis*													
Strain		1	2	3	4	5	6	7	8	9	10	11	12
1	NpR20-16 (AP038937, AP038938)		84.7	84.5	78.8	97.2	79.2	80.1	82.2	81.9	80.0	80.0	80.1
2	NpR20-75 (AP038939, AP038940)	27.7		100	78.7	84.8	79.4	80.1	82.9	81.9	79.9	79.9	79.9
3	NpR20-97 (AP038941, AP038942)	27.7	100		78.8	84.7	79.4	80.1	82.9	81.9	79.9	80.0	80.0
4	*Methylocystis bryophila* H2s^T^ (GCA_002117405.1)	21.6	21.6	21.6		79.0	78.7	78.3	78.6	78.7	78.4	78.6	78.4
5	*Methylocystis echinoides* LMG 27198^T^ (AP038927–AP038932)	74.4	27.8	27.8	22.0		79.2	80.0	82.6	82.2	80.0	79.9	80.1
6	*Methylocystis heyeri* H2^T^ (GCA_004802635.2)	22.1	21.3	21.3	21.4	21.7		78.7	79.2	78.8	78.3	78.3	78.5
7	*Methylocystis hirsuta* CSC1^T^ (GCA_003722355.1)	22.2	22.2	22.2	21.5	22.2	21.9		79.9	79.8	91.9	94.1	93.7
8	*Methylocystis iwaonis* SS37A-Re^T^ (GCA_027925385.1)	24.5	25.2	25.2	21.7	24.9	22.5	22.0		82.2	80.0	80.0	79.8
9	*Methylocystis parva* OBBP^T^ (GCA_027571405.1)	23.9	23.8	23.8	21.3	24.3	21.2	21.7	24.8		79.7	79.8	79.6
10	*Methylocystis rosea* SV97^T^ (GCA_000372845.1)	22.0	22.0	22.0	21.5	22.0	20.8	47.2	22.3	21.6		92.4	91.6
11	*Methylocystis silviterrae* FS^T^ (GCA_013350005.1)	21.9	21.9	21.9	21.0	21.8	21.1	56.0	21.8	21.7	49.1		93.5
12	*Methylocystis suflitae* NLS-7^T^ (GCA_024448135.1)	22.3	22.3	22.3	20.9	22.1	20.9	54.1	22.0	21.9	46.1	53.7	

(c) *Methylosinus*										
Strain		1	2	3	4	5	6	7	8	9
1	NpR20-40 (AP038943–AP038948)		97.2	98.9	97.2	97.1	99.8	98.9	97.0	82.9
2	NpR20-52 (AP038949–AP038953)	74.5		97.3	100	98.3	97.3	97.2	97.6	83.0
3	NpR20-53 (AP038954–AP038958)	89.4	75.6		97.3	97.2	99.0	99.8	97.2	83.1
4	NpR20-67 (AP038959–AP038962)	74.3	99.9	75.4		98.3	97.2	97.2	97.5	83.0
5	NpR20-85 (AP038963–AP038967)	73.3	84.2	74.7	83.9		97.1	97.2	97.5	83.3
6	NpR21-114 (AP038968–AP038972)	98.8	74.4	89.8	74.1	73.4		98.9	97.0	83.0
7	TuR21-B3a (AP038973–AP038975)	89.9	74.6	97.8	74.3	73.7	89.8		97.1	83.0
8	*Methylosinus sporium* 5^T^ (GCA_003113265.1)	73.0	77.9	73.5	77.5	76.5	73.4	73.6		83.1
9	*Methylosinus trichosporium* OB3b^T^ (GCA_002752655.1)	25.5	25.3	25.6	25.4	25.8	25.6	25.5	25.4	
