# Supplementary material for: Expanding the Eco-collection of Methane-oxidizing Bacteria Inhabiting Rice Roots: Cultivation, Isolation, and Genomic Characterization of Isolates
Source: Microbes Environ. 2025 Oct 11;40(4):ME25012. doi: 10.1264/jsme2.ME25012 (PMC12727199; doi:10.1264/jsme2.ME25012)
Supplement: Supplementary file 1 — Supplementary Material [file 40_25012_s1.pdf]

Table S1. Number of MOB cultures and strains.

Method	Direct spread plate						Enrichment cultivation						Total
Year	2020			2021			2020			2021			Total
Cultivar	Np	Mu	Tu	Np	Mu	Tu	Np	Mu	Tu	Np	Mu	Tu	
Number of cultures	4	n.e.	n.e.	2	0	3	56	n.e.	n.e.	7	11	17	100
Strain	0	n.e.	n.e.	1	0	1	8	n.e.	n.e.	0	2	0	12

n.e., not examined; Np, Nipponbare; Mu, Muha; Tu, Tupa 121-3

Table S2. Information about purification of MOB strains.

Strain	MuR21-B4b	MuR21-B4c	NpR20-16	NpR20-75	NpR20-97	NpR20-40	NpR20-52	NpR20-53	NpR20-67	NpR20-85	NpR21-114	TuR21-B3a
Collected year of roots sample	2021	2021	2020	2020	2020	2020	2020	2020	2020	2020	2021	2021
Method	Enrichment culture	Enrichment culture	Enrichment culture	Enrichment culture	Enrichment culture	Enrichment culture	Enrichment culture	Enrichment culture	Enrichment culture	Enrichment culture	Direct spread plate	Direct spread plate
Temperature for cultivation	25°C	25°C	30°C	25°C	25°C	25°C	25°C	25°C	25°C	25°C	25°C	25°C
Number of purification steps	5	4	4	2	4	2	2	6	7	5	4	7
Final isolation method	Dilution-to-extinction	Colony isolation	Dilution-to-extinction	Colony isolation	Dilution-to-extinction	Colony isolation	Colony isolation	Dilution-to-extinction	Colony isolation	Colony isolation	Dilution-to-extinction	Colony isolation

Table S3. Summary of tools used for whole genome analysis in this study.

Strain	Related species	Sequencer	Library preparation kit	Binding kit	Assembler
MuR21-B4b	*Methylomonus koyamae*	Sequel II	SMRTbell Express Template Prep Kit v2.0	Sequel II Binding kit v2.0	Canu v2.0
MuR21-B4c	*Methylomonas koyamae*	Sequel II	SMRTbell Express Template Prep Kit v2.0	Sequel II Binding kit v2.0	SMRTLink v10.2
NpR20-16	*Methylocystis echinoides*	Sequel II	SMRTbell Express Template Prep Kit v2.0	Sequel II Binding kit v2.0	SMRTLink v10.2
NpR20-75	*Methylocystis echinoides*	Sequel II	SMRTbell Express Template Prep Kit v2.0	Sequel II Binding kit v2.0	SMRTLink v10.2
NpR20-97	*Methylocystis echinoides*	Sequel II	SMRTbell Express Template Prep Kit v2.0	Sequel II Binding kit v2.0	SMRTLink v10.2
NpR20-40	*Methylosinus sporium*	Sequel II	SMRTbell Express Template Prep Kit v2.0	Sequel II Binding kit v2.0	SMRTLink v10.2
NpR20-52	*Methylosinus sporium*	Revio	SMRTbell Express Template Prep Kit v2.0	Revio polymerase kit	SMRTLink v13
NpR20-53	*Methylosinus sporium*	Sequel II	SMRTbell Express Template Prep Kit v2.0	Sequel II Binding kit v2.0	SMRTLink v10.2
NpR20-67	*Methylosinus sporium*	Revio	SMRTbell Express Template Prep Kit v2.0	Revio polymerase kit	SMRTLink v12
NpR20-85	*Methylosinus sporium*	Sequel II	SMRTbell Express Template Prep Kit v2.0	Sequel II Binding kit v2.0	Canu v2.0
NpR21-114	*Methylosinus sporium*	Sequel II	SMRTbell Express Template Prep Kit v2.0	Sequel II Binding kit v2.0	Canu v2.0
TuR21-B3a	*Methylosinus sporium*	Sequel II	SMRTbell Express Template Prep Kit v2.0	Sequel II Binding kit v2.0	Canu v2.0
Methylocystis echinoides LMG 27198^T	*Methylocystis echinoides*	Sequel II	SMRTbell Express Template Prep Kit v2.0	Sequel II Binding kit v2.0	Canu v2.0

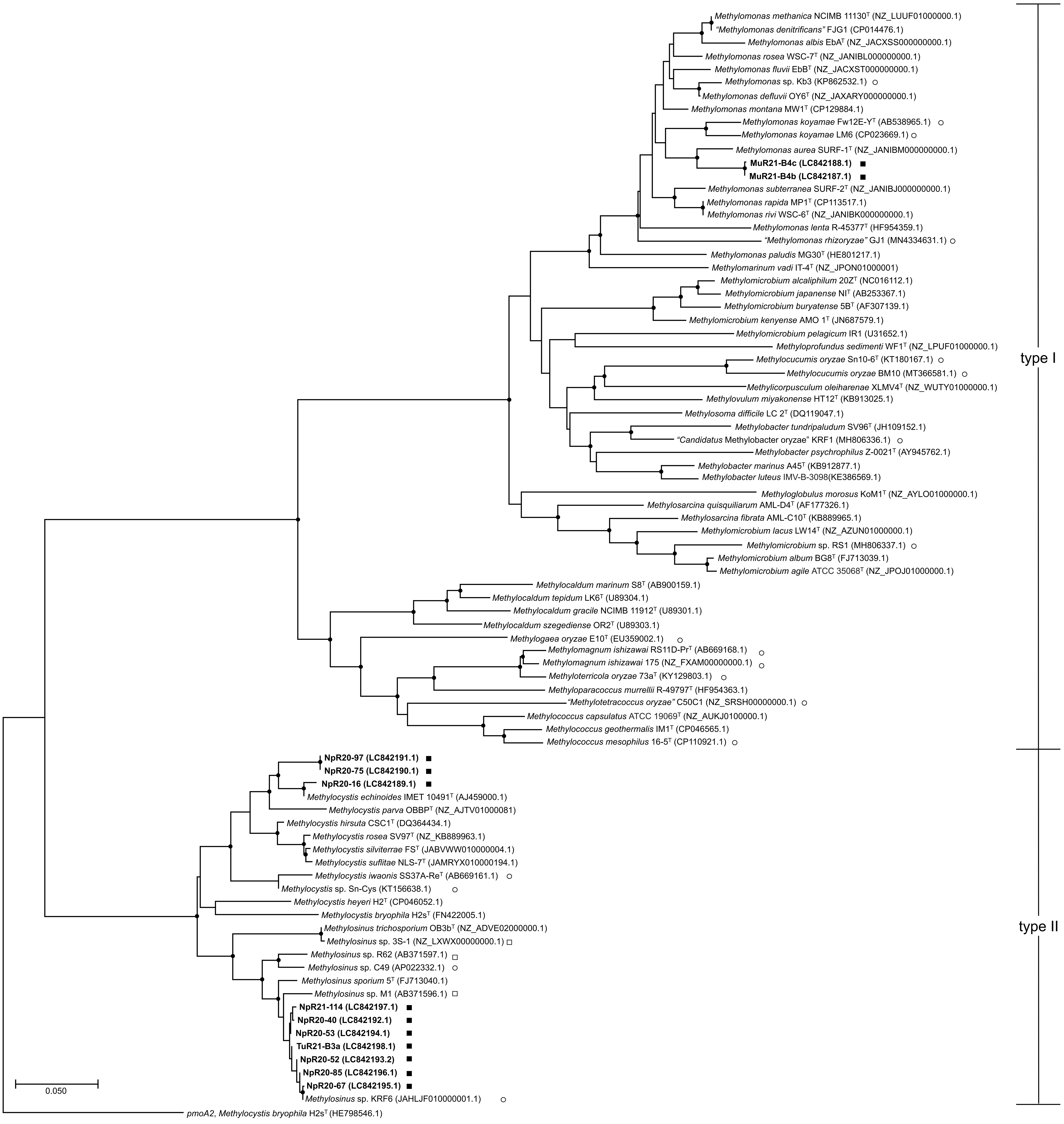

Fig. S1. Neighbor-joining phylogenetic tree based on *pmoA* gene sequences of MOB strains. Closed square, isolates from rice roots in this study; open square, isolates from rice roots in previous studies; open circle, isolates from rice fields other than rice roots. Bar, 0.05 substitutions per nucleotide sequence position. Filled circles indicate internal nodes with at least 50% bootstrap support from 1000 data resamplings. The tree was rooted using *pmoA2* gene of *Methylocystis bryophila* H2s^{T} as the outgroup. GenBank accession numbers are given in parentheses.

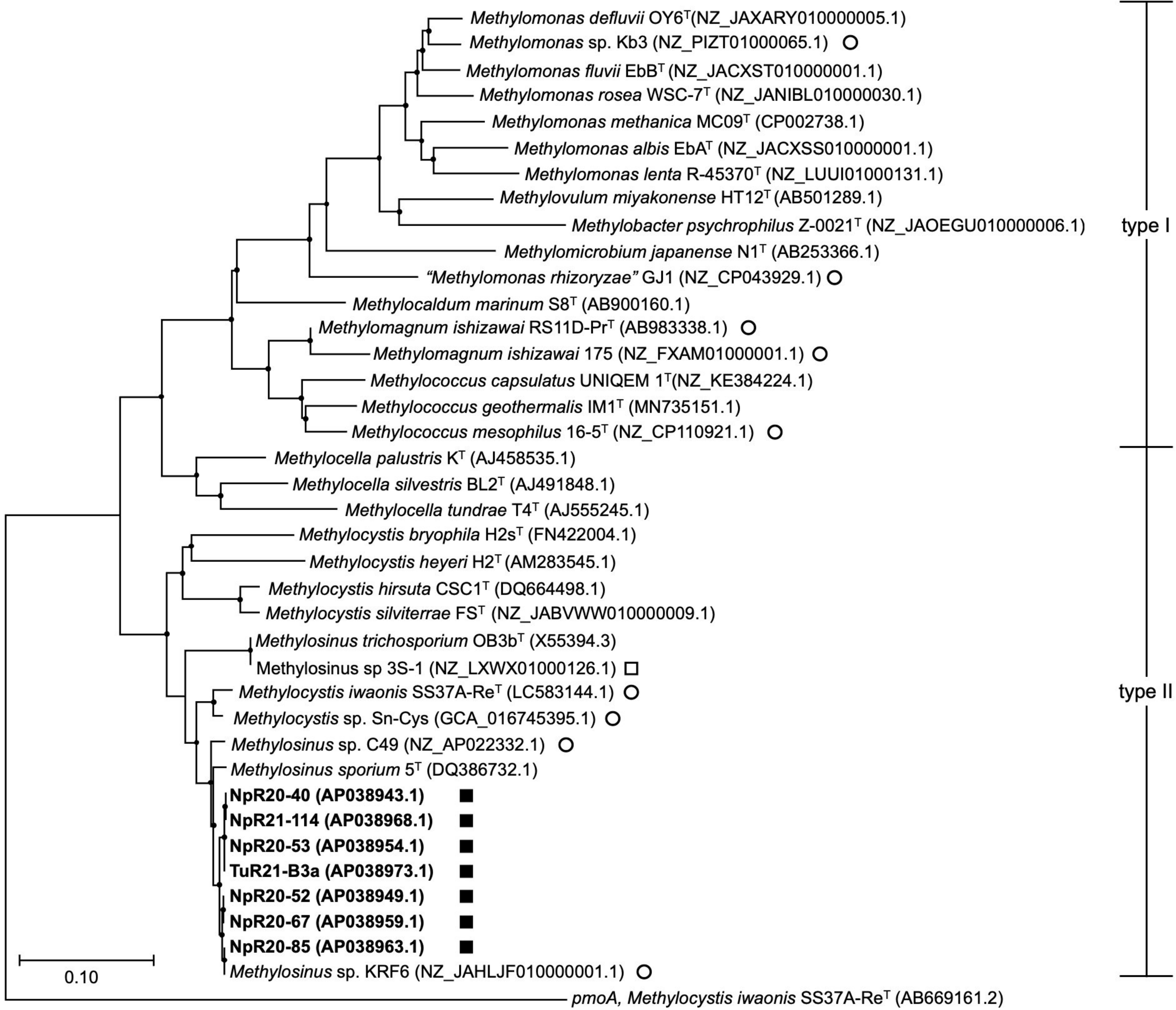

Fig. S2. Neighbor-joining phylogenetic tree based on *mmoX* gene sequences of MOB strains. Closed square, isolates from rice roots in this study; open square, isolates from rice roots in previous study; open circle, isolates from rice fields other than rice roots. Bar, 0.05 substitutions per nucleotide sequence position. Filled circles indicate internal nodes with at least 50% bootstrap support from 1000 data resampling. The tree was rooted using *pmoA* gene of *Methylocystis iwaonis* SS37A-Re^{T} as the outgroup. Accession numbers are given in parentheses.

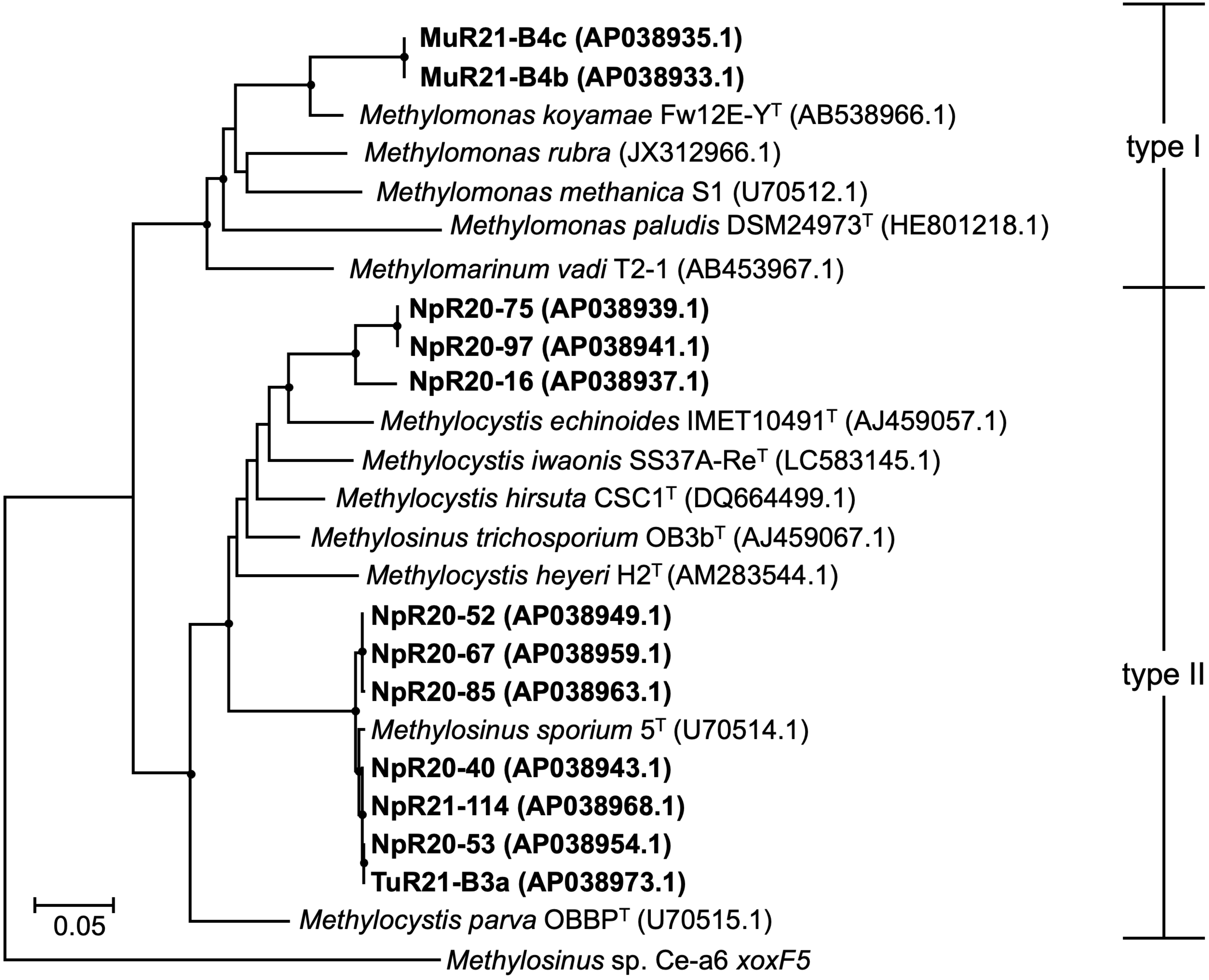

Fig. S3. Neighbor-joining phylogenetic tree based on *mxaF* gene sequences of MOB strains. Bar, 0.05 substitutions per nucleotide sequence position. Filled circles indicate internal nodes with at least 50% bootstrap support from 1000 data resampling. The tree was rooted using *xoxF5* gene of *Methylosinus* sp. Ce-a6 the outgroup. Accession numbers are given in parentheses.

(a)

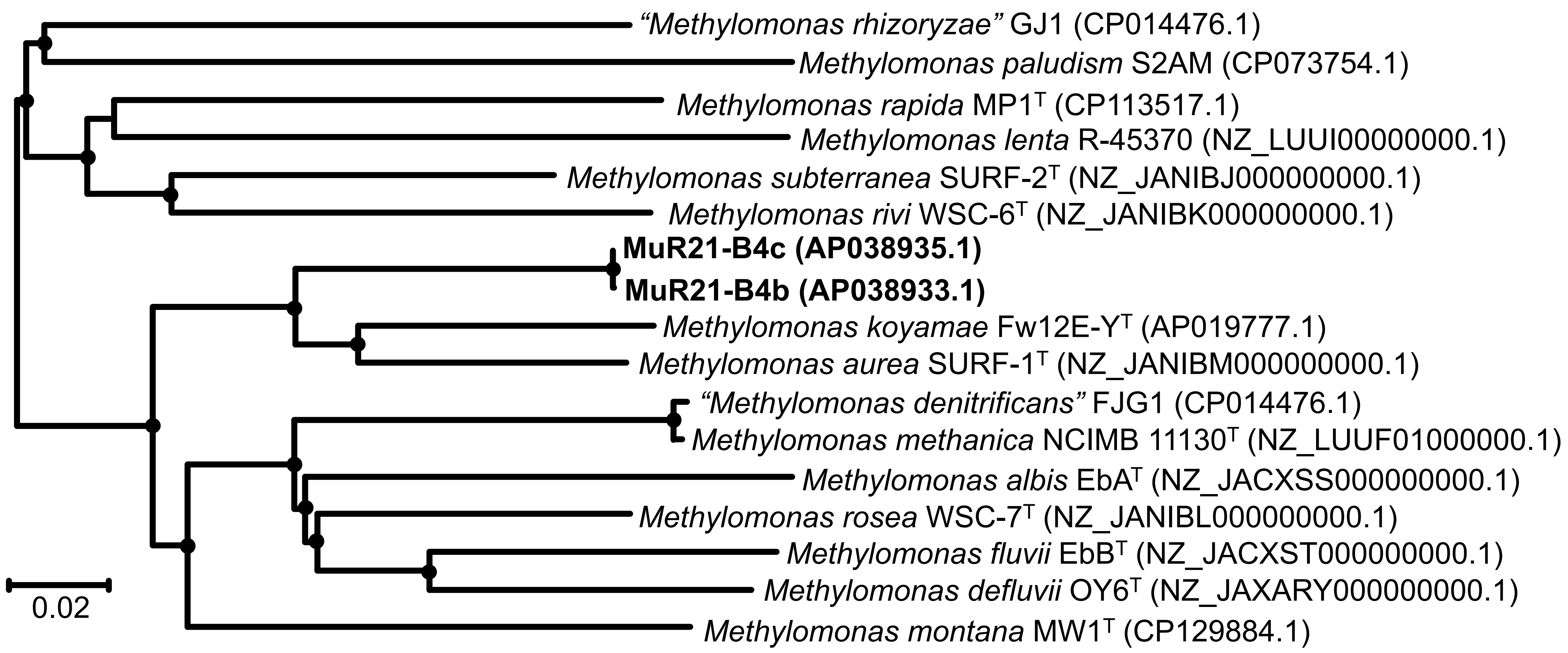

(b)

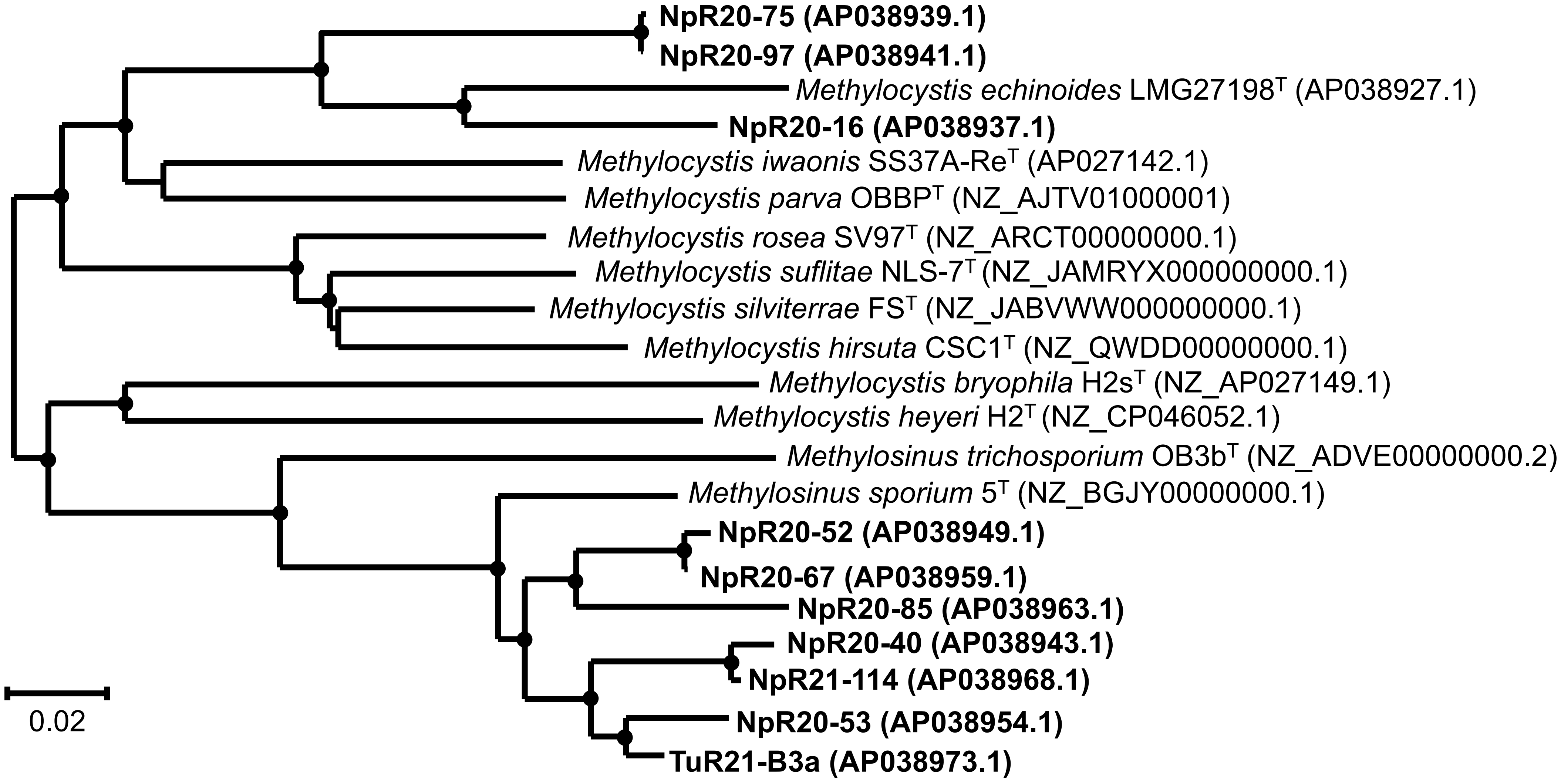

Fig. S4. Phylogenomic tree based on the concatenated nucleotide sequences of core genes of species of the genera (a) *Methylomonas*, and (b) type II MOB (*Methylocystis* and *Methylosinus*) by the distance method. Bar, 0.02 nucleotide substitutions per site. Filled circles indicate internal nodes with at least 70 % bootstrap support from 1000 data resamplings.
